# Health economic analysis of total extraperitoneal repair *versus* Lichtenstein surgery for inguinal hernia: data from a randomized clinical trial

**DOI:** 10.1093/bjsopen/zrab026

**Published:** 2021-04-08

**Authors:** L Westin, G Sandblom, U Gunnarsson, U Dahlstrand

**Affiliations:** Department of Trauma and Reparative Medicine, CLINTEC, Karolinska Institute, Karolinska University Hospital, Sweden; Department of Clinical Science and Education Södersjukhuset, Karolinska Institute, Sweden; Department of Surgery, Södersjukhuset, Sweden; Department of Surgical and Perioperative Science, Division of Surgery, Umeå University, Sweden; Department of Surgery, CLINTEC, Karolinska Institute, Enköping Hospital, Sweden

## Abstract

**Background:**

The aim was to compare cost-effectiveness of Lichtenstein under local anaesthesia (LLA) with total extraperitoneal repair (TEP) under general anaesthesia for primary inguinal hernia in men. An endoscopic approach to inguinal hernia repair is often considered costlier. The cost of endoscopic hernia repair, however, has not been compared to open inguinal hernia repair in a cost-effective setting.

**Methods:**

Data from an RCT comparing TEP and Lichtenstein in a cost-effective setting, with health economy as a secondary endpoint, were used. Data on costs were collected prospectively. Data on sick leave were obtained from the Swedish Social Insurance Agency in order to compare lengths of sick leave.

**Results:**

In total, 384 patients were included and 374 (97.4 per cent) patients were available for analysis, 189 in the LLA group and 185 in the TEP group. The median operating time for LLA was 70 (i.q.r. 60–80) min compared with 60 (i.q.r. 50–75) min in the TEP group (*P* < 0.001). The median time in operating theatre was 114 (i.q.r. 95–-125) min for LLA and 125 (i.q.r. 110–145) min for TEP (*P* < 0.001). The median cost including all materials was 2433 (i.q.r. 2084–2734) Euros for LLA and 2395 (i.q.r. 2093–2784) Euro for TEP (*P* = 0.650). Mean sick leave was 4.2 days in the LLA group and 6.2 days in the TEP group (*P* = 0.830).

**Conclusion:**

The overall cost to the hospital or length of sick leave did not differ between LLA and TEP.

## Teaser

This study is a cost-optimized comparison of TEP and Lichtenstein with costs as the primary outcome. From this RCT we concluded that there was no difference in costs for these two procedures and that cost does not need to be a factor when choosing a surgical technique.

Overall costs are similar for laparoscopic versus open repair of inguinal hernia.

## Introduction

Inguinal hernia repair is one of the most common procedures in general surgery. In Sweden about 16 000 inguinal hernia repairs are performed each year^1^. Most of these patients are active and form part of the workforce.

In recent years, the main focus of improvement in inguinal hernia surgery has shifted from prevention of hernia recurrence to prevention of postoperative complications, in particular chronic pain. This shift has resulted in the evolution of alternative surgical techniques. Laparoscopy has been shown to be beneficial in other types of surgery, especially regarding postoperative pain, wound infection and recovery time^2^. Laparoscopic groin hernia repair may either be performed as transabdominal preperitoneal repair (TAPP) or total extraperitoneal repair (TEP). International guidelines have recently been updated and make recommendations based on what type of patient and hernia is involved^3^. The two methods most frequently performed in Sweden today are the Lichtenstein technique and TEP.

Studies have shown the laparoscopic technique to be superior to Lichtenstein in some respects, including postoperative pain, recovery time and foreign body sensation^4^^,^^5^. However, severe complications, such as visceral organ injury, have been reported with laparoscopic repair^6^^,^^7^.

Furthermore, studies have suggested that the learning curve for the laparoscopic technique is longer^7^^,^^8^. This, combined with the suggestions that laparoscopy would be costlier, is a relevant concern within the surgical profession, since the cost aspect is rapidly becoming a major consideration in daily practice.

Although there are several studies comparing the cost of the open Lichtenstein technique with laparoscopic repair, none of these have been performed in a cost-effective setting^9^. Most studies compare several surgical techniques using different forms of anaesthesia. This makes it difficult to evaluate true cost.

The aim of this study was to compare the costs of the two prevailing methods in Sweden, TEP and Lichtenstein, in a cost-effective setting using prospectively recorded data from an RCT.

## Method

### Study design and participants

The current study was a cost analysis based on data from an RCT^4^. The RCT was designed to include data suitable for a health economic analysis, defining cost and sick leave as secondary outcome measures. In this study, the authors performed an analysis to compare the lengths of sick leave for the two surgical techniques. The trial was performed in accordance with the CONSORT criteria for RCTs^10^.

Patients were included in the RCT between 10 April 2006 and 5 January 2011. Inclusion criteria were men aged 20–80 years with a unilateral primary inguinal hernia. Exclusion criteria were: female gender; age below 20 or above 80 years; ASA physical status score IV or above; bilateral hernias; scrotal hernia; recurrent hernia; and previous surgery in the lower abdomen (except appendectomy). Recruitment and treatment of the patients was carried out at the two hospitals in one county in Sweden. The study was approved prior to the first enrolment by the Regional Ethics Committee in Uppsala, Sweden, and is registered with ClinicalTrials.gov, number NCT01020058.

### Randomization

**Fig. 1:**
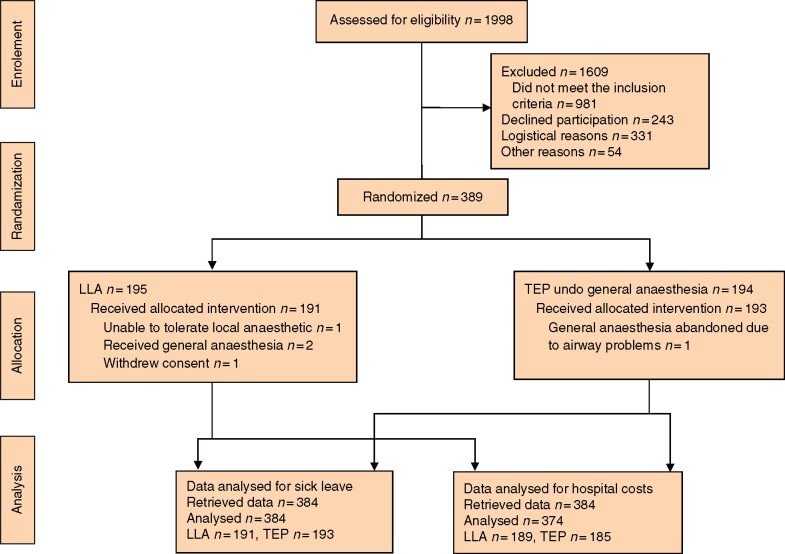
Flow chart for the study LLA, LLA Lichtenstein under local anaesthesia; TEP, total extraperitoneal repair

Block randomization on a 1 : 1 ratio between Lichtenstein under local anaesthesia (LLA) and TEP under general anaesthesia was performed (*Fig. 1*). Patients were randomized to LLA or TEP by the operating surgeon on the day of surgery. The complete randomization and masking procedure has been described previously^4^.

### Procedures

All operations were planned as outpatient procedures regardless of randomization. The four participating surgeons were experienced in both surgical techniques at the time of the trial and did not have a personal preference for either method. Heavyweight polypropylene mesh was used in all procedures.

Patients allocated to LLA underwent surgery according to the standard Lichtenstein technique^11^. Non-absorbable monofilament suture was used for fixation of the mesh. The local anaesthetic, a mixture 1 : 1 of bupivacaine 5 mg/ml and mepivacaine 10 mg/ml, was administered by the surgeon^12^. Any extra medication, such as sedation, was recorded.

In the TEP procedure, no dissection balloon and only reusable instruments were used. In this approach, non-fixation of the mesh was recommended. The decision to use staples or glue was made by the surgeon depending on the anatomical circumstances. If so, this was recorded, as was the use of any other equipment not routinely used.

In the LLA group, the repair was performed under local anaesthesia in accordance with previous studies showing the optimal cost-effectiveness of this technique for open inguinal hernia surgery^13^. In the TEP group costs were reduced by the use of reusable instruments, no fixation and no dissection balloon. The two procedures were thus performed in a cost-effective setting.

The following variables were registered: operating time; operating room (OR) time; materials used; and drugs administered. The definition of operating time was the time from start to finish of surgery. The materials used and their costs are specified in *Table 1*. Data on operating time and anaesthesia time were retrieved from the hospitals’ databases and cross-checked with the data recorded at the time of surgery. The cost per minute of operating time and OR time were 23.61 Euros and 5.95 Euros respectively.

**Table 1:** Standard and extra material used and the costs

Total extraperitoneal repair	Lichtenstein under local anaesthesia
Sutures: 1 Monocryl 3-0 (3 Euros) 1 PDS 2-0 (3 Euros)	Sutures: 1 Monocryl 3-0 (3 Euros) 1 PDS 2-0 (3 Euros) 1 Prolene 2-0 (2 Euros)
Heavyweight polypropylene mesh (62 Euros)	Heavyweight polypropylene mesh (62 Euros)
Standard laparoscopic reusable instruments (25 Euros)	Standard hernia instruments (25 Euros)
Diathermy (31 Euros)	Diathermy (31 Euros)
3 small surgical bandages (3 Euros/piece) Extra material: Tackers (226 Euros), skin stapler (3 Euros), surgical glue (13 Euros), suction and irrigation (25 Euros)	1 large surgical bandage (3 Euros)

Materials and instruments used for both procedures according to the study protocol. Extra materials such as glue or tackers were registered when used. Prices rounded to the nearest euro.

Episodes of sick leave within 1 year after surgery were recorded for all patients. This was made possible using the unique personal identification numbers given to all individuals registered as living in Sweden^14^^,^^15^. All patients were identified in the Försäkringskassan registry (the Swedish Social Insurance Agency).

### Outcomes

The outcomes in this study were direct cost to the healthcare system and length of sick leave as an indication of potential costs on society. The actual costs were retrieved in Swedish Krona (SEK) and then converted to Euros according to the average exchange rate for 2018 (1 SEK = 0.0975 Euros).

### Statistical analysis

Statistical analyses were made using Stata/IC version 12.1 (StataCorp, College Station, Texas, USA). Mann–Whitney *U* test was applied generally, since the distributions of the measured variables did not follow a normal distribution. The power analysis in the original study was performed for the primary outcome, postoperative pain. An estimate of power for the present secondary endpoint was therefore checked post hoc to determine the risk of missing a true difference. With 80 per cent power to detect a 10 per cent difference in the total cost to the hospital, a sample of 160 patients, 80 patients in each group, was required. The authors therefore concluded that with the present sample, a significant difference in cost should be detected.

Sick leave episodes were excluded if registered with an ICD-10 diagnosis code considered irrelevant for the surgical procedure or if registered before the date of hernia surgery. The ICD codes considered eligible for analysis are presented in *Table 2*. These criteria were applied to ensure that only sick leave related to the surgical procedure was analysed. For patients with part-time sick leave, the percentage of registered sick leave was used to calculate the corresponding full-time sick leave, which was then used in the analysis.

**Table 2:** Diagnoses, according to ICD-10, considered relevant for inclusion in analysis of sick-leave

ICD	Frequency (n = 87)
K40 (inguinal hernia)	82 (94)
M79 (other soft tissue disorders, not elsewhere classified)	3 (3)
R10 (abdominal and pelvic pain)	1 (1)
R52 (pain, not elsewhere classified)	1 (1)

Values in parentheses are percentages. ICD (International Statistical Classification of Diseases and Related Health Problems) codes accepted and analysed for sick leave analysis.

## Results

In all, 389 male patients with primary unilateral inguinal hernia were included. In five cases the protocol was violated during administration of anaesthesia and these patients were thus excluded from the study prior to the surgical intervention, leaving 384 patients who underwent surgery according to the study allocation. After randomization, 193 patients were allocated to TEP and 191 to LLA. The baseline characteristics of the two patient groups are presented in *Table 3*.

**Table 3:** Baseline patient characteristics

Characteristic	TEP	LLA	*P* ^†^
Age (years)*	52.9 (23–79)	53.2 (27–77)	0.980
Mean BMI (kg/m²)	26.5	24.9	0.310
ASA classification			
ASA I	124 (66.0)	134 (71.7)	0.230^‡^
ASA II	62 (33.0)	50 (27.3)	0.230^‡^
ASA III	2 (1.1)	2 (1.1)	1.000^§^

unless otherwise indicated. Values in parentheses are percentages

* Values are mean (range). TEP, total extraperitoneal repair; LLA, Lichtenstein under local anaesthesia.

† Mann–Whitney *U* test, except

‡ χ^2^-test,

§ Fischer’s exact test.

Ten patients were subsequently excluded since it was not possible to verify the data regarding costs for these patients in the hospital database. This left 374 (97.4 per cent) patients for final analysis, 189 patients in the LLA group and 185 patients in the TEP group.

The median operating time for LLA was 70 (i.q.r. 60–80) min and for TEP 60 (i.q.r. 50–75) min (*P* < 0.001). Median OR time was 114 (i.q.r. 95–125) min in the LLA group and 125 (i.q.r. 110–145) min in the TEP group (*P* < 0.001). The median total cost for the time a patient was in the operating theatre was 2329 Euros for the LLA group and 2159 Euros for the TEP group (*P* = 0.014). All differences between the groups were thus statistically significant.

The cost of material for the two standardized procedures was calculated to be 129 Euros and 134 Euros for LLA and TEP respectively, a difference of 5 Euros. When calculating the total cost of time in the operating theatre, material costs including extra materials used, the median cost became 2433 (i.q.r. 2084–2734) Euros for LLA and 2395 (i.q.r. 2092–2784) Euros for TEP (*P* = 0.650).

Registry data from the Swedish Hernia Register showed seven complications during the first 30 days after surgery. These were minor complications such as seroma and haematoma. There was one intraoperative complication in each group recorded in the national register. Both were minor vascular complications, resolved by electrocautery and ligature, respectively. In addition to this, the authors’ own registration of intraoperative complications also included three cases of peritoneal tearing requiring action in the TEP group. Within the follow-up time of 1 year there were two recurrences in the TEP group and four in the LLA group.

The study protocol dictated that patients should not stay overnight. However, two patients in the LLA group and six patients in the TEP group had to be admitted after surgery with a total of 3 and 8 in-patient days respectively in the groups.

All 384 originally included patients were registered by Swedish Social Insurance Agency. Data on sick leave during the first postoperative year were retrieved for all patients. The mean(s.d.) sick leave for relevant diagnoses (*Table 2*) was 4.2(9.40) days for LLA and 6.2(27.21) days for TEP (*P* = 0.830). A total of 85 patients were granted sick leave: 38 (19.5 per cent) patients in the LLA group and 47 (24.2 per cent) patients in the TEP group. When performing a χ^2^ test for whether patients had had any sick leave or not there was no significant difference (*P* = 0.260). In this population 13 patients were retired, six in the LLA group and seven in the TEP group.

## Discussion

Total costs for TEP and Lichtenstein did not differ in this RCT where each repair was performed in an optimal cost-effective setting. Although open as well as laparoscopic groin hernia repair merit their status as routine methods for different indications, cost-effectiveness *per se* cannot be used as an argument against any approach as long as it is performed in the most cost-effective setting possible.

Contrary to previous studies, the operating time for TEP was not longer than for the Lichtenstein repair^16^^,^^17^. These results are in accordance with the most recent studies showing that the operating time for laparoscopic repair performed at high-volume units is shorter. This indicates that the phase in the learning curve of the surgeon must be considered when assessing the outcome of laparoscopic methods. The present study was based on four surgeons who had passed their learning curve for TEP. The learning curve has a greater impact on laparoscopic repair operating time than it does on the time taken for LLA, and this would affect the results and the external validity of any other study not taking this factor into account^5^. Since the surgeons in this study were equally at ease with both techniques, this was not an issue.

The present study also found that LLA, as expected, had a significantly shorter anaesthetic time. The anaesthetic for the LLA was administered by the surgeon performing the procedure. Since the anaesthetic was administered in the operating theatre, this contributed to the OR time and may have affected the result. Today it is common for the surgeon to administer the local anaesthetic before the patient is taken into the theatre. This is even more efficient and probably more cost-effective. It could be argued that surgery under local anaesthesia does not necessarily need to be performed in an operating theatre with all the infrastructural requirements for general anaesthesia, and that costs thus could be further reduced for these procedures. Such a practice would limit the group of eligible patients and might introduce both higher cost and logistical problems in cases where conversion to general anaesthesia is necessary.

The results from this study are only applicable to male patients that are medically suitable for either LLA or TEP. This will exclude women and patients with bilateral hernias for example, where endoscopic repair may be preferred. However, the majority of patients will still fall within the criteria that were applied to the study population. The recurrence rate was fairly low; however, it is known that this risk increases with time and could therefore have a somewhat larger impact after several years.

When duration of surgery as well as OR times and materials were included in the analysis, the costs for LLA and TEP did not differ significantly. This is in contrast to the study by McCormack and colleagues who showed a significantly lower cost for Lichtenstein repair^9^. In the present model, operating time was considerably more expensive per minute (23.61 Euros per minute) than anaesthetic time (5.95 Euros per minute). Since LLA took a median of 10 minutes longer operating time, representing 244 Euros, this would explain the lack of difference in cost between TEP and LLA in the present study.

The study does not include the anaesthetic drugs used; this is a weakness as it does not provide the complete costs for the procedures. The authors attempted to include these costs but were unable to derive the consumption and costs of these drugs.

The sick leave analysis in this study did not reveal a difference between the groups. However, the study can only report the time of sick leave and not the actual cost. Very few sick leave days were registered, the analysis is based on all 384 patients and the means did not surpass 1 week. Both groups seem generally to have recovered and returned to their normal activity level within a few days. In this population only 13 patients were retired. Due to the exclusion criteria, the mean age of the patients in the study was somewhat lower than that recorded in the Swedish Hernia Registry. To be able to analyse the true economic effects for society of the sick leave, the study would have had to include a model to calculate the expected incomes of the individual patients and the frequency of lost income. This would have given a more complete overview of the economic effects.

From these results, the authors conclude that there was no significant difference in direct cost between TEP and LLA. Laparoscopic repair, however, has the advantage of reducing the risk for persisting pain, which is still a major issue, and is useful in cases where Lichtenstein repair is less appropriate due to anatomical or technical reasons. There are factors that warrant the choice of either TEP or LLA for hernia repair, but cost should not be one of them.

## Funding

The study was funded by Uppsala-Örebro Regional Research Council, Stockholm County Council, the Swedish Society of Medicine and the Olle Engqvist Research Foundation.

*Disclosure*. The authors declare no conflict of interests..
